# Post-Transplant Maintenance Therapy in Acute Myeloid Leukemia

**DOI:** 10.3390/cancers16112015

**Published:** 2024-05-26

**Authors:** Katherine Parks, Muhammad Faisal Aslam, Vinod Kumar, Omer Jamy

**Affiliations:** 1 Department of Medicine, University of Alabama at Birmingham, Birmingham, AL 35294, USA; 2 St. Vincents Hospital, Birmingham, AL 35294, USA; 3 Division of Hematology & Oncology, Department of Medicine, University of Alabama at Birmingham, 1720 2nd Avenue S, NP2540W, Birmingham, AL 35294, USA

**Keywords:** acute myeloid leukemia, post-transplant, maintenance therapy

## Abstract

**Simple Summary:**

The leading cause of failure of allogeneic hematopoietic cell transplantation (allo-HCT) in patients with acute myeloid leukemia (AML) is post-transplant relapse. We urgently need strategies to decrease this risk. In the past few years, the basic framework of post-transplant maintenance has been shaped by several clinical trials investigating targeted, immunomodulatory, and cellular therapies. Although the practice of post-transplant maintenance in AML has become more common, there remain challenges regarding the feasibility and efficacy of this strategy. In this review, we discuss recent advances in post-transplant maintenance in AML, along with ongoing and future planned studies in this area, outlining the limitations of available data and our future goals.

**Abstract:**

Allogeneic hematopoietic cell transplantation (allo-HCT) is potentially curative for patients with acute myeloid leukemia (AML). However, the post-transplant relapse rate ranges from 40 to 70%, particularly with reduced intensity conditioning, and remains a major cause of treatment failure for these patients due to the limited efficacy of salvage therapy options. Strategies to mitigate this risk are urgently needed. In the past few years, the basic framework of post-transplant maintenance has been shaped by several clinical trials investigating targeted therapy, chemotherapy, and immunomodulatory therapies. Although the practice of post-transplant maintenance in AML has become more common, there remain challenges regarding the feasibility and efficacy of this strategy. Here, we review major developments in post-transplant maintenance in AML, along with ongoing and future planned studies in this area, outlining the limitations of available data and our future goals.

## 1. Introduction

Acute myeloid leukemia (AML) is a heterogeneous group of blood cell cancers that arise from the clonal expansion of malignant hematopoietic precursor cells in the bone marrow, which contributes to the aggressive nature of this cancer [1]. The prognosis varies based on age, comorbidities, and cytogenic and mutation status [1,2,3]. Treatment is initiated with standard induction chemotherapy to induce a complete remission (CR) which will be followed by consolidation chemotherapy or allogeneic hematopoietic stem cell transplantation (allo-HCT) depending on disease risk based on cytogenetic and molecular aberrations at presentation and response to therapy assessed by measurable residual disease (MRD) at the time of transplant, donor availability, comorbidities, age, and performance status [1,2,3]. If the patient is determined to have intermediate- or adverse-risk AML, allo-HCT is strongly recommended. Despite its central role in the management of AML, only a minority of patients for whom transplantation is indicated undergo the procedure due to biological factors, personal and physician choice, and lack of access. Even when these patients undergo transplantation, relapse after allo-HCT remains the leading cause of failure. Previously, non-relapse mortality related to allo-HCT was a significant barrier to survival for patients with AML, but recently, non-relapse mortality has decreased significantly due to higher-resolution HLA typing, less toxic conditioning regimens, improved anti-infectious agents, and novel approaches to graft-versus-host disease (GVHD) prevention and treatment [2,4,5]. Unfortunately, the risk of relapse has not seen the same degree of reduction over the past several years. Now, the focus of recent research has shifted to investigating different approaches to prevent relapse after allo-HCT since around 20–30% of patients with AML will relapse in the first 1–2 years following allo-HCT and a shorter time to relapse is correlated with worse outcomes [1,2,3,6]. After relapse, the prognosis is poor, and the median survival is only 4–6 months, with a 1-year survival rate of less than 20% [1,2,3,6]. These figures represent the importance of identifying strategies to decrease rates of relapse and improve post-transplant survival. There are currently three general approaches to therapy after allo-HCT, which include maintenance therapy, preemptive therapy, and therapy for active relapse disease [1,2]. The goal of maintenance therapy is to deliver a minimally toxic therapy that can reduce the risk of relapse before any signs of MRD develop, but there continue to be challenges regarding the feasibility and efficacy of this strategy. We will review major developments in post-transplant maintenance therapy for AML, along with ongoing and future planned studies in this area, outlining the limitations of available data and future goals.

## 2. Who Should Receive Post-HCT Maintenance Therapy?

Before initiation of maintenance therapy, it is important to consider who is at higher risk for relapse and if the risks of maintenance therapy outweigh the potential benefits. Patients that are at an increased risk for relapse include those with high-risk disease based on cytogenetic/molecular abnormalities, presence of pre- or post-HCT MRD, active disease at the time of transplant, and those receiving reduced intensity conditioning regimens [1]. In addition to considering who is at high risk for relapse, it is important to recognize those patients for whom the risk from maintenance therapy will outweigh the potential benefit. Patients with significant post-HCT complications, such as delayed hematopoietic recovery or ongoing toxicities such as GVHD, infection, or organ dysfunction, can be at increased risk for adverse effects of maintenance therapy [2]. Furthermore, the toxicity profile of the therapeutic agent may also dictate the decision to offer maintenance therapy. As the side effect profile varies from targeted maintenance options to non-targeted ones, the ability to administer maintenance agents depends on the patient’s ongoing clinical situation as well as their ability to specifically handle that agent. Thus, it is important to consider all these factors for each patient and appropriately select patients at high risk for relapse before initiating maintenance therapy. Research surrounding this topic has increased greatly in recent years with an expanding number of available therapeutic agents, such as hypomethylating agents (HMAs), HMA/BCL-2 inhibitors, FLT3 inhibitors, IDH inhibitors, HDAC inhibitors, glasdegib, eprenetapopt, checkpoint inhibitors, and cellular therapy including donor lymphocyte infusion (DLI), which will be discussed in detail below.

### 2.1. Key Issues of Maintenance Therapy

Maintenance therapy focuses on indiscriminately treating all indicated patients before the development of MRD or frank relapse, in order for them to remain in remission. Inevitably, maintenance therapy potentially over-treats a significant number of patients who otherwise may not have needed additional therapy, exposing the patient to the risk of unnecessary toxicities [1,2]. One of the main focuses of current research is identifying an effective agent with a low toxicity profile to mitigate this risk.

Important factors to consider for post-HCT maintenance therapy include determining the optimal time to initiate maintenance therapy post-transplant. Sometimes, this decision can be made easier with the presence of a sensitive assay for MRD such as the FLT3 assay used in the MORPHO trial with a level of detection of 1 × 10^−6^ [7]. Unfortunately, such sensitive assays are not that readily available for most other genotypes in AML. Additionally, the duration of maintenance therapy and the financial burden of a potentially long-term treatment are important to clearly define. Also, understanding and outlining the potential drug–drug interactions that can occur between the maintenance therapy regimen and other drugs such as antifungal azoles, calcineurin inhibitors, and mTOR inhibitors will allow for safe administration [1]. In addition, the immunocompromised state after allo-HCT can lead to the presence of active opportunistic infections, which complicates the initiation of post-HCT maintenance therapy. Lastly, any agent being considered for maintenance needs to be extensively investigated to ensure that it does not result in an increased risk of GVHD. Consequently, these factors emphasize the need for well-designed, randomized clinical trials to characterize the benefits and practicalities of various maintenance therapy agents. Another consideration is the utilization of murine and canine animal models, which have long served to help develop tolerable conditioning regimens for elderly patients as well as effective prevention and treatment of GVHD. These models can potentially be leveraged to prevent relapse by gaining insight into graft-versus-leukemia (GVL) [8]. Murine hematological cancer cell lines, cultured in vitro, are sensitive to alloreactive T-cell targeting, in contrast to human tumors [9]. However, spontaneous tumors in companion dogs have overlapping features with tumors in humans, and there are reports describing autologous transplantation as consolidation therapy for canine lymphoma [8,10]. Other reports demonstrate the feasibility of generating canine leukemia models through genetic modification of canine hematopoietic stem cells prior to transplant [8]. With these models, there is the potential to understand both the efficacy and toxicity of agents to be used for post-transplant maintenance.

### 2.2. Hypomethylating Agents

Hypomethylating agents such as azacitidine (AZA) and decitabine (DEC) are nucleoside analogues that irreversibly bind to enzymes responsible for methylation and induce cellular apoptosis [3]. This class of drugs has been successful in treating patients with AML who are unable to tolerate high-intensity regimens, such as older, medically non-fit patients, because of their favorable toxicity profile. Additionally, this class of drugs may potentially help to eradicate MRD, decrease the incidence of GVHD, and facilitate the graft-versus-leukemia effect by enhancing the effect of T-regulatory lymphocytes. However, these benefits of HMAs have not been consistently shown across studies and need further investigation [1,2,3,6]. In the setting of a favorable toxicity profile and perhaps with the possibility of facilitating the eradication of remaining AML cells, HMAs have been used for post-transplant maintenance therapy to prevent relapse.

Initially, in 2010, De Lima et al. conducted a phase I trial including 45 high-risk patients with AML/MDS to determine if low-dose AZA could be a safe and effective post-transplant maintenance therapy. This study determined that AZA 32 mg/m^2^ was well tolerated, with reversible thrombocytopenia as the most common adverse effect. Regarding efficacy, this study demonstrated a 1-year event-free survival (EFS) of 58% and 1-year overall survival (OS) of 77% [11]. The RELAZA2 trial was an open-label, multicenter, phase II trial that demonstrated the effectiveness of AZA at preventing or substantially delaying hematologic relapse in MRD-positive patients with MDS or AML. In this study, AZA at 75 mg/m^2^/day on days 1–7 of a 29-day cycle resulted in a 2-year OS of 62% and 2-year EFS of 54% [12]. This regimen was well tolerated, with neutropenia as the most common side effect [12]. De lima et al. investigated the utilization of CC-486 for AML/MDS patients in morphologic CR at 42 to 84 days after allo-SCT in a phase I/II dose finding study. This study demonstrated that CC-486 (oral AZA) maintenance was generally well tolerated, with low rates of relapse, disease progression, and GVHD, and a randomized phase III trial comparing CC-486 to placebo as post-transplant maintenance is currently ongoing (AMADEUS, NCT04173533) [13]. Oran et al. conducted a phase III randomized control trial to investigate the efficacy and safety of AZA maintenance in post-transplant settings based on the encouraging phase I and II reports for AZA maintenance in patients with AML/MDS. A total of 187 patients were enrolled to receive either AZA (32 mg/m^2^ every 4 weeks for 12 cycles) or a placebo. The median number of cycles administered was four. The median relapse-free survival (RFS) was 2.07 years in the AZA group vs. 1.28 years in the control group, and there was no significant difference in overall survival. The incidence of both acute and chronic GVHD was comparable between the two groups. Additionally, there were no unexpected adverse events observed in patients receiving AZA. A substantial number of adverse events occurred in the control arm, which were related to typical post-transplant complications, and myelosuppression was the only toxicity directly related to AZA treatment. Although the benefit of HMA therapy as post-transplant maintenance was not evident in this study, the authors did note many patients were unable to complete the planned number of cycles and that perhaps the lower dose of AZA might be insufficient to improve transplant outcomes, emphasizing the need for further investigation in maintenance strategies [14].

DEC is another hypomethylating agent used in induction chemotherapy in AML/MDS patients who are unable to tolerate intensive chemotherapy regimens. There has been evidence that DEC could also be used as a maintenance therapy for post-HCT patients [15]. Gao et al., in a randomized phase II trial, demonstrated that the combination of G-CSF and DEC administered every 6 to 8 weeks for a total of six courses, when compared to placebo, resulted in a statistically significant reduction in the risk of relapse without increasing the risk of chronic GVHD [16]. Pusic et al. led a phase I trial including 22 patients with AML/MDS in CR after allo-HCT to investigate four different DEC dose levels (5, 7.5, 10, and 15 mg/m^2^/day every 6 weeks for a maximum of eight cycles) at the same frequency; although lower doses were associated with decreased hematologic toxicities, relapse was seen in 6 of the 22 patients [15]. From 2015 to 2018, Ma et al. conducted a retrospective study to evaluate the use of DEC 20 mg/m^2^/day for 5 days every 3 months as post-HCT maintenance therapy, which showed enhanced graft-versus-leukemia effects, reduced GVHD, and prevented relapse compared to the lower-dose DEC used in Pusic et al. [15,17].

Although many studies have reported on post-HCT maintenance with HMAs with variable results, the feasibility and efficacy of this strategy should still be considered investigational given the lack of clear survival benefit and the challenges of completing therapy due to the side effect profile [11,12,13,15,17]. There is a need for additional randomized clinical trials to investigate the different HMA doses and schedules, oral HMAs, and combinations of other agents with HMAs to determine a clear conclusion regarding improvement in outcomes in AML patients post-HCT. Several such studies are ongoing.

### 2.3. Hypomethylating Agents + BCL-2 Inhibitors

Venetoclax (VEN), a highly selective BCL-2 inhibitor, in combination with an HMA, is safe and tolerable in patients unfit to receive intensive chemotherapy [18]. BCL-2 is an oncoprotein that prevents apoptosis, thus promoting cell survival; therefore, a BCL-2 inhibitor aims to inactivate the oncoprotein and allow for cellular death [3]. VEN has been shown to possess significant anti-leukemic activity, help mitigate the risk of GVHD, and work synergistically with HMAs against AML [19]. Kent et al. investigated VEN 400 mg daily, as monotherapy, for maintenance therapy and noted promising findings of 1-year post-HCT OS of 70% and RFS of 67% with the most common adverse effects including cytopenia and gastrointestinal toxicities. There was no increased risk of GVHD observed with this strategy [20]. Parks et al. investigated low-dose DEC at 10 mg/m^2^ for 3 days and Ven 200 mg as post-HCT maintenance therapy, which showed a 1-year non-relapse mortality of 11%, 1-year OS of 84%, manageable cytopenias, and no increased risk of GVHD in 26 patients with high-risk myeloid malignancies [21].

Wei Y et al. determined that low-dose DEC 15 mg/m^2^ for 3 days and VEN 200 mg on days 1–21 every 2 months for up to 10 cycles after allo-HCT can be administered safely, with reversible hematologic toxicities, neutropenic fever, and fatigue as the most common side effects and no evidence of increased incidence of GVHD. Additionally, this combination had a 1-year relapse incidence of 15.3%, 2-year OS of 85.2%, and 2-year EFS of 84.7% [22]. Garcia et al. conducted a phase I trial that showed that VEN/AZA maintenance therapy after VenFluBu2 RIC allo-HCT appeared to be safe, with a low infection rate and no excessive GVHD [23]. The optimal duration of maintenance with the combination of HMA/VEN is not clear, but most studies have aimed for administering 8 to 12 cycles given every 4 to 8 weeks. Although early data appear promising, the degree of myelosuppression observed with this combination, when used for the treatment of AML, may be a barrier for patients in the early post-transplant period. Lastly, the VIALE-T study is an ongoing randomized, open-label, phase III study evaluating the safety and efficacy of VEN/AZA versus placebo after allo-HCT in patients with acute myeloid leukemia [19]. Results from this study are eagerly awaited and will help provide insight into the potential benefit as well as risk associated with post-transplant HMA/VEN maintenance strategies. Interestingly, the VIALE-T study investigates the combination of VEN/AZA administered every 28 days for 6 cycles followed by VEN monotherapy until up to 24 cycles.

### 2.4. FLT3 Inhibitors

The most commonly found molecular aberrations in AML are FLT3 mutations. FLT3-ITD (internal tandem duplication) is present in 25% of adults and FLT3-TKD mutations constitute 10% of AML cases [24]. FLT3-ITD mutation causes activation of tyrosine kinase leading to increased activity of receptor tyrosine kinase (RTK) and is associated with increased risk of relapse and lower survival. Both sorafenib and gilteritinib have been incorporated in the clinic as post-HCT maintenance for relapse prevention. Smaller studies with midostaurin and quizartinib have also been conducted.

Gilteritinib is indicated as monotherapy for the treatment of relapsed/refractory (r/r) FLT3-mutated AML. It was compared to placebo as post-transplant maintenance in BMT-CTN1506, a randomized phase III trial. The primary endpoint of the trial was RFS. The trial utilized a FLT3 MRD assay with a level of detection of 1 × 10^−6^. Gilteritinib was administered at 120 mg for 24 months post-transplant. The trial enrolled 356 patients. More than half the patients did receive FLT3 inhibitors as pre-transplant therapy. For the entire cohort, gilteritinib was associated with a higher RFS but the difference was not statistically significant (HR, 0.679, 95% CI, 0.459 to 1.005; two-sided *p* = 0.0518). In a subgroup analysis, patients with pre- or post-transplant MRD (50.5%) derived a significant benefit in RFS from gilteritinib (HR = 0.515, 95% CI:0.316, 0.838, *p* = 0.0065) whereas those in MRD-negative remissions did not. Additionally, geographical differences were also observed, with patients in North America deriving benefit from maintenance but those in Europe and Asia/the rest of the world not seeing an advantage of the strategy. Possible reasons for these regional variations may include time to transplant, the type of pre-transplant therapy including the use FLT3 inhibitors, and the type of conditioning regimen. Gilteritinib was associated with more myelosuppression, which was also the major cause of early withdrawal for the study. This study was helpful in highlighting the importance of MRD-directed intervention and establishing FLT3-ITD as a potential marker for MRD [7].

Sorafenib, an RTK inhibitor, inhibits FLT3 by directly blocking auto-phosphorylation. Post-transplant sorafenib was studied in a phase I trial where patients with FLT3-mutated AML received a variety of conditioning regimens [25]. Twenty-two patients were enrolled, and the maximum tolerated dose (MTD) was determined to be 400 mg BID. Five patients stopped sorafenib due to toxicities that included weight loss, tongue/facial swelling, persistent nausea, diarrhea, and other gastrointestinal symptoms. The 1-year PFS was 85% (90% CI, 66–94%) and the OS was 95% (90% CI, 79–99%). Although sorafenib was well tolerated at an MTD of 400 mg BID, FLT3 inhibition was appreciated at a lower well-tolerated dose of 200 mg BID as well [25].

In the phase II SORMAIN trial, 83 patients were randomized to receive sorafenib (n = 43) and placebo (n = 40) as post-transplant maintenance. The primary endpoint of the study was RFS. Within the limitations of a small sample size, the use of sorafenib was associated with a lower risk of relapse and death in patients with FLT3-ITD-mutated AML. A third of the patients were beyond first remission at the time of transplant [26].

In a phase III trial, 202 patients with FLT3-ITD-mutated AML undergoing allo-HCT were randomized to maintenance therapy with either sorafenib 400 mg BID (n = 100) or placebo (n = 102) [27] for up to 6 months post-transplant. The primary endpoint was 1-year cumulative incidence of relapse. With a median follow-up of 60 months, sorafenib was associated with improved OS (72·0% [95% CI 62·1–79·7] vs. 55·9% [45·7–64·9]; hazard ratio [HR] 0·55, 95% CI 0·34–0·88; *p* = 0·011), improved leukemia-free survival (70·0% [60·0–78·0] vs. 49·0% [39·0–58·3]; 0·47, 0·30–0·73; *p* = 0·0007), lower relapse risk (15·0% [8·8–22·7] vs. 36·3% [27·0–45·6]; 0·33, 0·18–0·60; *p* = 0·0003), and no increase in non-relapse mortality (15·0% [8·8–22·7] vs. 14·7% [8·6–22·3]; 0·79, 0·39–1·62; *p* = 0·98). There was no difference in the incidence of GVHD between the two groups, and the most common grade III-IV adverse events associated with sorafenib were infection and hematological toxicities. Longer follow-up did not reveal any difference in late side effects with sorafenib [27].

In a small phase II trial (RADIUS), the use of midostaurin as post-transplant maintenance was well tolerated and did not result in an increased risk of GVHD. Common side effects were gastrointestinal toxicities, as is known from its pre-transplant use. The sample size was small (n = 60) and the study was not powered to detect treatment effects. Nonetheless, there was a trend towards benefit with midostaurin for all efficacy endpoints evaluated [28].

Quizartinib was recently approved for the treatment of AML with FLT3-ITD mutation. A study (QuANTUM-First) allowed for it to be used as maintenance therapy both pre- and post-transplant. Quizartinib resulted in a survival benefit compared to placebo [29]. In a phase I trial of quizartinib as post-transplant maintenance in 13 patients with FLT3-ITD-mutated AML, myelosuppression was the most common adverse event. One patient relapsed early in the trial. The study demonstrated acceptable tolerance of quizartinib as post-transplant maintenance [30]. Crenolanib, another FLT3i, is also being investigated for post-transplant maintenance (NCT02400255).

Based on published data, the use of FLT3 inhibitors to mitigate the risk of post-transplant relapse is recommended. The evidence is strongest for patients with any evidence of MRD in the peri-transplant period. For patients with MRD-negative disease, the decision to proceed with maintenance would incorporate other variables such as pre-transplant exposure to FLT3 inhibitors, co-mutations, and conditioning intensity. While characterizing the molecular landscape of FLT3-mutated AML, the presence of both NPM1 and DNMT3A confers a survival benefit with the use of post-transplant sorafenib maintenance [31]. Outcomes with gilteritinib were also favorable for ‘triple-mutated’ AML in the relapse setting [32]. Conversely, the presence of TP53 as well as co-occurring CEBPA mutations did not result in much benefit being derived from the addition of sorafenib in the post-transplant setting [31]. As we obtain more data from the post-transplant gilteritinib trial, we hope to be able to validate previous findings and perhaps identify further molecular subsets that may benefit from post-transplant maintenance. Lastly, the duration of FLT3i maintenance is not standardized, with 24 m of maintenance being the most studied schedule.

### 2.5. IDH Inhibitors

Isocitrate dehydrogenase 1 (IDH1) and IDH2 are two important enzymes that catalyze the decarboxylation of isocitrate to a-ketoglutarate (a-KG) in the Krebs cycle. IDH1 mutation is identified in approximately 5 to 10% of patients with AML whereas IDH2 is identified in 8 to 15% of patients [33,34]. Ivosidenib inhibits IDH1, while ensidenib targets IDH2. Both are FDA-approved for treatment of patients with AML harboring these mutations. Currently, these mutations are predictive but not prognostic, and therefore the true benefit of targeting them in the maintenance setting is unclear. Nonetheless, there have been studies investigating these agents in the maintenance setting.

Both these drugs have been investigated as post-transplant maintenance therapies, for up to 12 months, in multicenter phase I clinical trials [34,35]. Ivosidenib was studied at a standard dose of 500 mg and a second de-escalated dose of 250 mg if the higher dosing was intolerable. Sixteen out of eighteen enrolled patients received post-transplant ivosidenib. The drug was well tolerated, with grade III adverse events including QTc prolongation and paresthesia observed in two and one patients, respectively. Eight participants discontinued therapy before completing the predetermined 12 cycles: three due to relapse, three for treatment of cGVHD, one due to pruritis, and one due to patient preference. The dose of 500 mg was established as the recommended phase II dose (RP2D). The risk of GVHD was not increased with this strategy, and the 6-month incidence of gII-IV aGVHD was 6.3% and the 2-year incidence of all cases of cGVHD was 63%. The 2-year incidences of non-relapse mortality and relapse were 0% and 19%, respectively. With a median follow-up of 29 months, the 2-year PFS and OS were 81% and 88%, respectively [35].

Similarly, enasidenib was investigated at 50 mg and 100 mg as post-transplant maintenance in patients with AML/MDS with IDH2 mutation. Nineteen out of twenty-three enrolled patients received post-transplant enasidenib. The drug was well tolerated and no dose-limiting toxicities were observed. The dose of 100 mg was established as the RP2D. Grade III-IV toxicities were mainly hematological. Eight participants discontinued therapy before completing the predetermined 12 cycles: three due to toxicities, two for treatment of GVHD, one due to relapse, one due to physician choice, and one due to COVID-19. The risk of GVHD was not increased with this strategy, with no cases of gIII-IV aGVHD and a 12 month incidence of moderate/severe cGVHD of 42%. The cumulative incidence of relapse was 16%. The 2-year PFS and OS were 69% and 74%, respectively [34].

Both these studies highlight the feasibility of introducing AML treatment drugs as post-transplant maintenance strategies. Within the limitations of a small sample size, the strategy appears to be well tolerated and perhaps even somewhat effective. These results merit additional investigation of these agents as both monotherapies as well as combination therapies in larger studies. Given that the combined prevalence of IDH1/2 is up to 25%, these studies require multi-institutional collaboration.

### 2.6. Potential Combination Therapies

Whereas epigenetic inhibitors can potentially prevent post-transplant relapse through various immunomodulatory properties inhibiting the evasion of AML cells from the immune system, it is essential to understand the heterogeneity of AML as a disease. The impact of different molecular aberrations, combined with the role of MRD, on treatment effects needs to be investigated to improve maintenance strategies [36]. In this context, we discuss some combinations of the drugs discussed previously. Of note, all these combinations were investigated in the pre-transplant setting but may provide valuable information on both efficacy and toxicity for transplant recipients.

Enasidenib + venetoclax: Preclinical studies have shown that IDH2-mutated cells are sensitive to BCL-2 inhibition. In a phase Ib/II trial of 27 patients with r/r IDH2-mutated AML, the combination of enasidenib and venetoclax was well tolerated, with common grade III-V adverse events being febrile neutropenia (41%), thrombocytopenia (26%), and hyperbilirubinemia (15%). The CR rate was 57% and the median survival was 9.4 months, encouraging further investigation [37].

Ivosidenib + venetoclax + azacitidine: The triplet combination resulted in a composite remission rate of 87% in patients with r/r IDH1-mutated AML. Common grade III-V toxicities mainly included neutropenia (29%). Nearly half of the participants required dose adjustments for myelosuppression. The combination might overcome established resistance pathways to the single agent ivosidenib and is undergoing further evaluation [38].

Gilteritinib + venetoclax: Preclinical models have demonstrated synergy between gilteritinib and venetoclax in FLT3-mutated AML. In a phase Ib trial, the most common grade III-IV adverse event was cytopenias (80%), requiring dose interruptions in 50% of the patients. The composite response was 75% and the combination was effective in a population previously exposed to FLT3 inhibitors. Nonetheless, measures were needed to mitigate myelosuppression, and further studies are ongoing [39].

These doublets and triplets are being actively investigated in larger trials in the pre-transplant setting. Response rates are high, but we also observe bone marrow suppression. Randomized trials will tell us if combination therapy outperforms the standard of care in the pre-transplant setting. If it does, we then need to be cautious in our approach to utilizing these agents as maintenance therapies given that there is a lot unknown about interactions with grafts, immunosuppressive agents, and infection prophylaxis.

### 2.7. Glasdegib

The Hedgehog (Hh) signaling pathway is involved in the maintenance, growth, and drug-resistance mechanisms of both MDS and AML [40]. Therapy targeting the Hh pathway signaling can eliminate leukemic stem cell populations and play an important role in the treatment of disease resistance. Glasdegib is a new drug class that inhibits this pathway. Glasdegib and low-dose cytarabine (LDAC) have been approved for the treatment of AML in specific populations that cannot tolerate intensive therapy [41]. Glasdegib monotherapy has also been investigated as post-transplant maintenance therapy for patients with AML and MDS to prevent relapse. In a dual-center pilot study, 31 patients (28 AML and 3 MDS) were administered glasdegib 100 mg as post-transplant maintenance for up to 1 year in the absence of relapse. Glasdegib was associated with adverse events impacting the quality of life of the participants, and this led to both interruption and discontinuation of therapy. The incidence of relapse at 1 year was 55%, and the authors concluded that glasdegib did not result in a meaningful reduction in the risk of post-transplant relapse. Further studies of glasdegib in select patient populations as well as combination therapy could be helpful [42].

### 2.8. Histone Deacetylase Inhibitors

Histone deacetylase inhibitors (HDACi) are epigenetic modifiers and have shown significant effects in the differentiation and apoptosis of myeloid cells. Trials have been carried out that have shown their moderate anti-leukemic effects in select patients with advanced AML and MDS [43]. In a phase I/II trial of 42 patients receiving post-transplant panobinostat maintenance, the estimated 2-year OS and RFS were 81% and 75%, respectively. Grade III-IV toxicities included myelosuppression, gastrointestinal symptoms, neurological symptoms, and constitutional symptoms, and nearly all resolved with drug interruption. The risk of both acute and chronic GVHD was not increased with the use of panobinostat [44]. Vorinostat is currently under evaluation in a phase I trial (NCT03843528) in combination with low-dose azacitidine.

### 2.9. APR246 (Eprenetapopt)

AML and MDS harboring TP53 mutations have dismal outcomes, even after proceeding to allo-HCT [45]. Hence, there is a need for targeted agents for this high-risk population. Eprenetapopt is a first-in-class, small molecule p53 re-activator that works by selectively inducing apoptosis of TP53 mutant cancer cells [46]. In a phase II trial, the combination of eprenetapopt and AZA, every 28 days for a maximum of 12 cycles, was investigated as post-transplant maintenance in patients with TP53-mutated AML or MDS. The primary endpoint was RFS. Thirty-three patients received maintenance therapy. With a median follow-up of 14.5 months, the estimated 1-year RFS was 59.9%, and with a median follow-up of 17 months the estimated 1-year OS was 78.8%. The combination did not result in an increased risk of GVHD. The most common adverse effects were hematologic toxicities and hypertension [46]. Unfortunately, the future of eprenetapopt is not clear given the negative results of a large phase III trial in high-risk MDS (NCT03745716).

### 2.10. Cellular Therapy

#### 2.10.1. Donor Lymphocyte Infusion (DLI)

The use of DLI has been extensively investigated over time. It has limited utility in the treatment of frank relapse and is more commonly utilized as a preemptive strategy for the emergence of MRD or loss of chimerism. The mechanism of action is based on non-tolerant T cells augmenting the graft-versus-leukemia effect but at the expense of causing GVHD [47]. Liga et al. investigated low-dose prophylactic DLI in 15 patients with acute leukemia treated with an alemtuzumab-containing conditioning regimen. Although none of the patients relapsed, there were four deaths due to severe GVHD, leading to the termination of the study [48]. In a registry study utilizing matched pair analysis, the administration of prophylactic DLI did not improve outcomes for the entire cohort of acute leukemia patients. However, a subset analysis demonstrated a survival benefit for patients with high-risk AML. Given the small numbers as well as the possibility of a selection bias given the long interval between transplant and DLI, the authors concluded that the efficacy of DLI to prevent relapse was moderate at best [49]. In a retrospective study, Jedlickova et al. found the 7-year OS to be 67% in patients with AML/MDS receiving escalating doses of DLI starting 120 days post-HCT. In the control group, the 7-year OS was only 31%. Along with the retrospective nature of the study, other major limitations included the exclusion of patients with a history of GVHD prior to the administration of DLI as well as the heterogeneity in the control arm [50]. In a phase II trial of 30 patients, the combination of low-dose AZA and DLI as prophylaxis for high-risk AML and MDS resulted in a 2-year OS of 65.5% without an increased risk of GVHD. The regimen was well tolerated but needs validation in larger studies [51]. The use of prophylactic DLI as monotherapy as well as in combination with chemotherapy is an active area of collaborative prospective trials [47].

#### 2.10.2. Natural Killer Cells (NK)

Leveraging NK cells to prevent post-transplant relapse is an area of active interest. Robust NK-cell donor chimerism and those with specific haplotypes of KIR genes (B-haplotype) may be associated with a reduced risk of post-transplant relapse. In a phase I trial, the use of prophylactic donor-derived ex vivo IL-2 activated NK cells in 16 patients with hematological malignancies, after matched sibling transplant, was well tolerated with no dose-limiting toxicities. Two patients developed moderate and one patient developed severe cGVHD which responded to corticosteroids. Four patients relapsed, and at the time of the last follow-up, eleven patients were alive, in remission, and without GVHD [52]. In a phase I/II trial, 25 patients with myeloid malignancies received high doses of mb-IL21 ex vivo expanded donor-derived NK cells, after haploidentical transplant, for relapse prevention. When compared to a case-matched cohort, the risk of relapse was lower in NK therapy recipients (4% vs. 38%, *p* = 0.014). One patient had grade III aGVHD and no patients developed cGVHD. Overall, the treatment was well tolerated [53]. These promising observations are early, and ongoing trials will provide more insight into the efficacy of this platform.

#### 2.10.3. Engineered T Cells

Donor-derived T-cell receptor engineered T cells against HA-1 and HA-2 (presented on HLA-A*02:01) have the potential to prevent relapse after transplant. In a phase I trial of TSC-100 and TSC-101 (engineered T cells) targeting HA-1/2 in recipients of reduced intensity haplo-transplant, there were no dose-limiting toxicities observed in seven patients. The risk of GVHD was not increased, and at the last update, there were no relapses or deaths after a median follow-up of 162 days [54]. This strategy appears attractive, and further studies are planned.

### 2.11. Immune Checkpoint Inhibitors (CPIs)

The use of CPIs as maintenance for patients with myeloid malignancies has also been investigated. Most of the studies have been conducted in a transplant-ineligible patient population [55]. Whereas CPIs might enhance the graft-versus-leukemia effect, there is a concern that they may also lead to an increased risk of immune-mediated adverse effects, mainly GVHD. When ipilimumab was used to treat 28 patients with relapsed hematological malignancies after allo-HCT, 5 had a complete response and 2 had a partial response. However, six patients had immune-related toxicities, including one death, and four patients had GVHD [56]. Similarly, an increased incidence of rapid and severe GVHD was also observed in patients with post-transplant relapsed lymphoid malignancies when treated with CPIs [57]. More recently, Zeiser et al. demonstrated that the use of sabatolimab, a novel immunotherapy targeting TIM-3, was well tolerated as post-transplant therapy in patients with AML MRD+ disease. In 21 patients, grade I-II myelosuppression was observed and there were no cases of GVHD or immune-related toxicities. Longer follow-up is needed to determine both the efficacy as well as the safety of sabatolimab [58].

## 3. Conclusions

Although the intention in proceeding with allo-HCT for patients with AML has always been to cure the disease, there remains a significant risk of post-transplant relapse. To mitigate this risk, there has been significant interest in the utilization of pre-transplant therapeutic agents as post-transplant maintenance. In this review, we have discussed the potential strategies that have been investigated (Table 1) and provided a list of active trials (Table 2). The challenges observed in conducting successful trials in the post-transplant setting include (1) the hesitancy of clinicians to enroll patients with high-risk disease in a placebo-controlled trial, even in the absence of approved agents, (2) the hesitancy of patients to continue to take chemotherapy after having undergone an intense and potentially curative procedure, (3) the complexity of delineating the additive toxicities of the investigational agent from common overlapping post-transplant toxicities, (4) the challenges of understanding how best to modify the dosing and schedule when using pre-transplant drugs in the post-transplant setting to maximize time on the drug and perhaps outcomes, (5) the ability to identify which patients will actually benefit from maintenance therapy, and (6) the reluctance of pharmaceutical companies to conduct maintenance trials prior to the approval of the agent for another indication.

Currently, the data support the use of FLT3 inhibitors such as sorafenib and gilteritinib, mainly in a risk-adaptive approach. Several other targeted agents, including BCL-2 and IDH inhibitors, and immunomodulatory/cellular strategies such as engineered T cells and NK-cell-based maintenance, appear promising in early studies but need to be tested in well thought out trials. Lessons from negative randomized trials include the use of MRD-directed maintenance to avoid exposing patients to unnecessary toxicity. Large, randomized trials for oral AZA as well as AZA/VEN have nearly completed accrual, and the data are eagerly awaited to facilitate our understanding of the role of maintenance therapy in AML and help design future trials for success.

## Figures and Tables

**Table 1:** Select published post-transplant maintenance trials in AML.

Agent (Phase)	Treatment	Findings	Clinical Trial Identifier
AZA (III)	AZA vs. placebo AZA 32 mg/m^2^ for 5 days	RFS: 2.07 years (AZA) vs. 1.28 years (placebo), *p* = 0.43 Myelosuppression with AZA	NCT00887068
DEC/VEN (I)	DEC 15 mg/m^2^ for 3 days Ven 200 mg days 1–21	1-year relapse = 15.3% 1-year OS = 85.2% Reversible myelosuppression, no increased risk of GVHD	ChiCTR1900025374
AZA/VEN (I)	AZA 36 mg/m^2^ for 5 days Ven 400 mg days 1–14	2-year relapse = 41% 2-year OS = 67% Reversible myelosuppression, no increased risk of GVHD	NCT03613532
Sorafenib (III)	Sorafenib 400 mg bid vs. placebo for 6 months	Improved 2-year RFS (78.9% vs. 56.6%) and OS (82.1% vs. 68.0%) with sorafenib No difference in GVHD No pre-transplant FLT3i	NCT02474290
Gilteritinib (III)	Gilteritinib 120 mg vs. placebo for 24 months	Improved RFS in MRD+ (HR = 0.515, 95% CI: 0.316, 0.838, *p* = 0.0065) with gilteritinib More myelosuppression with gilteritinib leading to early withdrawal	NCT02997202
Ivosidenib (I)	Ivosidenib 500 mg and 250 mg	2-year relapse = 19% 2-year PFS = 81% 2-year OS = 88% No increased risk of GVHD	NCT03564821
Enasidenib (I)	Enasidenib 100 mg and 50 mg	Relapse = 16% 2-year PFS = 69% 2-year OS = 74% No increased risk of GVHD	NCT03515512
APR246 (II)	APR246 + AZA in TP53-mutated AML	1-year RFS = 59.9% 1-year OS = 78.8% No increased risk of GVHD	NCT03931291

AZA: azacitidine, VEN: venetoclax, AML: acute myeloid leukemia, DEC: decitabine, GVHD: graft-versus-host disease, OS: overall survival, RFS: relapse-free survival, PFS: progression-free survival.

**Table 2:** Select active post-transplant maintenance trials in AML.

Trial	Treatment	Phase	Clinical Trial Identifier
AMADEUS	CC-486 vs. placebo	III	NCT04173533
VIALE-T	HMA/VEN vs. placebo	III	NCT04161885
Crenolanib	Single arm crenolanib maintenance in FLT3-AML	II	NCT02400255
Vorinostat	Vorinostat dose escalation after allogeneic hematopoietic cell transplantation	I	NCT03843528
Panobinostat	Panobinostat vs. placebo	III	NCT04326764

HMA: hypomethylating agent, VEN: venetoclax, AML: acute myeloid leukemia.

## References

[1] Al-Shaibani E., Novitzky-Basso I., Mattsson J., Kim D.D.H. (2023). Post-transplant maintenance therapy in acute myeloid leukemia after allogeneic hematopoietic stem cell transplantation harmonizing multiple therapeutic modalities including targeted therapy, immunotherapy and cellular therapy. Int. J. Hematol..

[2] DeFilipp Z., Chen Y.B. (2023). How I treat with maintenance therapy after allogeneic HCT. Blood.

[3] Manobianco S.A., Rakiewicz T., Wilde L., Palmisiano N.D. (2022). Novel Mechanisms for Post-Transplant Maintenance Therapy in Acute Myeloid Leukemia. Front. Oncol..

[4] Bolaños-Meade J., Hamadani M., Wu J., Al Malki M.M., Martens M.J., Runaas L., Elmariah H., Rezvani A.R., Gooptu M., Larkin K.T. (2023). Post-Transplantation Cyclophosphamide-Based Graft-versus-Host Disease Prophylaxis. N. Engl. J. Med..

[5] Jamy O., Zeiser R., Chen Y.B. (2023). Novel developments in the prophylaxis and treatment of acute GVHD. Blood.

[6] Kreidieh F., Abou Dalle I., Moukalled N., El-Cheikh J., Brissot E., Mohty M., Bazarbachi A. (2022). Relapse after allogeneic hematopoietic stem cell transplantation in acute myeloid leukemia: An overview of prevention and treatment. Int. J. Hematol..

[7] Levis M.J., Hamadani M., Logan B., Jones R.J., Singh A.K., Litzow M., Wingard J.R., Papadopoulos E.B., Perl A.E., Soiffer R.J. (2024). Gilteritinib as Post-Transplant Maintenance for Acute Myeloid Leukemia with Internal Tandem Duplication Mutation of FLT3. J. Clin. Oncol..

[8] Graves S.S., Parker M.H., Storb R. (2018). Animal Models for Preclinical Development of Allogeneic Hematopoietic Cell Transplantation. ILAR J..

[9] Stolfi J.L., Pai C.C., Murphy W.J. (2016). Preclinical modeling of hematopoietic stem cell transplantation-advantages and limitations. FEBS J..

[10] Deeg H.J., Appelbaum F.R., Weiden P.L., Hackman R.C., Graham T.C., Storb R.C. (1985). Autologous marrow transplantation as consolidation therapy for canine lymphoma: Efficacy and toxicity of various regimens of total body irradiation. Am. J. Vet. Res..

[11] de Lima M., Giralt S., Thall P.F., de Padua Silva L., Jones R.B., Komanduri K., Braun T.M., Nguyen H.Q., Champlin R., Garcia-Manero G. (2010). Maintenance therapy with low-dose azacitidine after allogeneic hematopoietic stem cell transplantation for recurrent acute myelogenous leukemia or myelodysplastic syndrome: A dose and schedule finding study. Cancer.

[12] Platzbecker U., Middeke J.M., Sockel K., Herbst R., Wolf D., Baldus C.D., Oelschlägel U., Mütherig A., Fransecky L., Noppeney R. (2018). Measurable residual disease-guided treatment with azacitidine to prevent haematological relapse in patients with myelodysplastic syndrome and acute myeloid leukaemia (RELAZA2): An open-label, multicentre, phase 2 trial. Lancet Oncol..

[13] de Lima M., Oran B., Champlin R.E., Papadopoulos E.B., Giralt S.A., Scott B.L., William B.M., Hetzer J., Laille E., Hubbell B. (2018). CC-486 Maintenance after Stem Cell Transplantation in Patients with Acute Myeloid Leukemia or Myelodysplastic Syndromes. Biol. Blood Marrow Transpl..

[14] Oran B., de Lima M., Garcia-Manero G., Thall P.F., Lin R., Popat U., Alousi A.M., Hosing C., Giralt S., Rondon G. (2020). A phase 3 randomized study of 5-azacitidine maintenance vs. observation after transplant in high-risk AML and MDS patients. Blood Adv..

[15] Pusic I., Choi J., Fiala M.A., Gao F., Holt M., Cashen A.F., Vij R., Abboud C.N., Stockerl-Goldstein K.E., Jacoby M.A. (2015). Maintenance Therapy with Decitabine after Allogeneic Stem Cell Transplantation for Acute Myelogenous Leukemia and Myelodysplastic Syndrome. Biol. Blood Marrow Transpl..

[16] Gao L., Zhang Y., Wang S., Kong P., Su Y., Hu J., Jiang M., Bai H., Lang T., Wang J. (2020). Effect of rhG-CSF Combined with Decitabine Prophylaxis on Relapse of Patients with High-Risk MRD-Negative AML after HSCT: An Open-Label, Multicenter, Randomized Controlled Trial. J. Clin. Oncol..

[17] Ma Y., Qu C., Dai H., Yin J., Li Z., Chen J., Qiu H., Sun A., Miao M., Fu C. (2020). Maintenance therapy with decitabine after allogeneic hematopoietic stem cell transplantation to prevent relapse of high-risk acute myeloid leukemia. Bone Marrow Transpl..

[18] DiNardo C.D., Jonas B.A., Pullarkat V., Thirman M.J., Garcia J.S., Wei A.H., Konopleva M., Döhner H., Letai A., Fenaux P. (2020). Azacitidine and Venetoclax in Previously Untreated Acute Myeloid Leukemia. N. Engl. J. Med..

[19] Craddock C., Platzbecker U., Heuser M., Pullarkat V., Chaudhury S., Wu D., Addo S., Chyla B., Jiang Q., Lee P. (2022). P561: VIALE-T: A randomized, open-label, phase 3 study of venetoclax in combination with azacitidine after allogeneic stem cell transplantation in patients with acute myeloid leukemia. HemaSphere.

[20] Kent A., Schwartz M., McMahon C., Amaya M., Smith C.A., Tobin J., Marciano K., Rezac R., Bosma G., Pollyea D.A. (2023). Venetoclax is safe and tolerable as post-transplant maintenance therapy for AML patients at high risk for relapse. Bone Marrow Transpl..

[21] Parks K., Diebold K., Salzman D., Di Stasi A., Al-Kadhimi Z., Espinoza-Gutarra M., Bhatia R., Jamy O. (2023). Low-dose decitabine plus venetoclax as post-transplant maintenance for high-risk myeloid malignancies. Blood.

[22] Wei Y., Xiong X., Li X., Lu W., He X., Jin X., Sun R., Lyu H., Yuan T., Sun T. (2021). Low-dose decitabine plus venetoclax is safe and effective as post-transplant maintenance therapy for high-risk acute myeloid leukemia and myelodysplastic syndrome. Cancer Sci..

[23] Garcia J.S., Kim H.T., Brock J., Murdock H.M., Cutler C.S., DeAngelo D.J., Gibson C.J., Gooptu M., Ho V., Koreth J. (2022). Maintenance Therapy with Venetoclax/Azacitidine Can be Safely Given after Venetoclax/FluBu2 RIC Allogeneic Transplantation for the Treatment of High Risk MDS/AML: Results of a Phase 1 Study. Blood.

[24] Zhao J.C., Agarwal S., Ahmad H., Amin K., Bewersdorf J.P., Zeidan A.M. (2022). A review of FLT3 inhibitors in acute myeloid leukemia. Blood Rev..

[25] Chen Y.B., Li S., Lane A.A., Connolly C., Del Rio C., Valles B., Curtis M., Ballen K., Cutler C., Dey B.R. (2014). Phase I trial of maintenance sorafenib after allogeneic hematopoietic stem cell transplantation for fms-like tyrosine kinase 3 internal tandem duplication acute myeloid leukemia. Biol. Blood Marrow Transpl..

[26] Burchert A., Bug G., Fritz L.V., Finke J., Stelljes M., Röllig C., Wollmer E., Wäsch R., Bornhäuser M., Berg T. (2020). Sorafenib Maintenance After Allogeneic Hematopoietic Stem Cell Transplantation for Acute Myeloid Leukemia With FLT3-Internal Tandem Duplication Mutation (SORMAIN). J. Clin. Oncol..

[27] Xuan L., Wang Y., Yang K., Shao R., Huang F., Fan Z., Chi P., Xu Y., Xu N., Deng L. (2023). Sorafenib maintenance after allogeneic haemopoietic stem-cell transplantation in patients with FLT3-ITD acute myeloid leukaemia: Long-term follow-up of an open-label, multicentre, randomised, phase 3 trial. Lancet Haematol..

[28] Maziarz R.T., Levis M., Patnaik M.M., Scott B.L., Mohan S.R., Deol A., Rowley S.D., Kim D.D.H., Hernandez D., Rajkhowa T. (2021). Midostaurin after allogeneic stem cell transplant in patients with FLT3-internal tandem duplication-positive acute myeloid leukemia. Bone Marrow Transplant..

[29] Erba H.P., Montesinos P., Kim H.-J., Patkowska E., Vrhovac R., Žák P., Wang P.-N., Mitov T., Hanyok J., Kamel Y.M. (2023). Quizartinib plus chemotherapy in newly diagnosed patients with <em>FLT3</em>-internal-tandem-duplication-positive acute myeloid leukaemia (QuANTUM-First): A randomised, double-blind, placebo-controlled, phase 3 trial. Lancet.

[30] Sandmaier B.M., Khaled S., Oran B., Gammon G., Trone D., Frankfurt O. (2018). Results of a phase 1 study of quizartinib as maintenance therapy in subjects with acute myeloid leukemia in remission following allogeneic hematopoietic stem cell transplant. Am. J. Hematol..

[31] Shao R., Zhang Y., He J., Huang F., Fan Z., Yang K., Xu Y., Xu N., Luo Y., Deng L. (2023). Impact of genetic patterns on sorafenib efficacy in patients with FLT3-ITD acute myeloid leukemia undergoing allogeneic hematopoietic stem cell transplantation: A multi-center, cohort study. Signal Transduct. Target Ther..

[32] Smith C.C., Levis M.J., Perl A.E., Hill J.E., Rosales M., Bahceci E. (2022). Molecular profile of FLT3-mutated relapsed/refractory patients with AML in the phase 3 ADMIRAL study of gilteritinib. Blood Adv..

[33] Murdock H.M., Ho V.T., Garcia J.S. (2024). Innovations in conditioning and post-transplant maintenance in AML: Genomically informed revelations on the graft-versus-leukemia effect. Front. Immunol..

[34] Fathi A.T., Kim H.T., Soiffer R.J., Levis M.J., Li S., Kim A.S., Mims A.S., DeFilipp Z., El-Jawahri A., McAfee S.L. (2022). Enasidenib as maintenance following allogeneic hematopoietic cell transplantation for IDH2-mutated myeloid malignancies. Blood Adv..

[35] Fathi A.T., Kim H.T., Soiffer R.J., Levis M.J., Li S., Kim A.S., DeFilipp Z., El-Jawahri A., McAfee S.L., Brunner A.M. (2023). Multicenter Phase I Trial of Ivosidenib as Maintenance Treatment Following Allogeneic Hematopoietic Cell Transplantation for IDH1-Mutated Acute Myeloid Leukemia. Clin. Cancer Res..

[36] Yan Y., Upadhyaya R., Zhang V.W., Berg T. (2022). Epigenetic maintenance strategies after allogeneic stem cell transplantation in acute myeloid leukemia. Exp. Hematol..

[37] Richard-Carpentier G., Gupta G., Cameron C., Cathelin S., Bankar A., Davidson M.B., Gupta V., Maze D.C., Minden M.D., Murphy T. (2023). Final Results of the Phase Ib/II Study Evaluating Enasidenib in Combination with Venetoclax in Patients with IDH2-Mutated Relapsed/Refractory Myeloid Malignancies. Blood.

[38] Lachowiez C.A., Loghavi S., Zeng Z., Tanaka T., Kim Y.J., Uryu H., Turkalj S., Jakobsen N.A., Luskin M.R., Duose D.Y. (2023). A Phase Ib/II Study of Ivosidenib with Venetoclax ± Azacitidine in IDH1-Mutated Myeloid Malignancies. Blood Cancer Discov..

[39] Daver N., Perl A.E., Maly J., Levis M., Ritchie E., Litzow M., McCloskey J., Smith C.C., Schiller G., Bradley T. (2022). Venetoclax Plus Gilteritinib for FLT3-Mutated Relapsed/Refractory Acute Myeloid Leukemia. J. Clin. Oncol..

[40] Cortes J.E., Gutzmer R., Kieran M.W., Solomon J.A. (2019). Hedgehog signaling inhibitors in solid and hematological cancers. Cancer Treat Rev..

[41] Cortes J.E., Douglas Smith B., Wang E.S., Merchant A., Oehler V.G., Arellano M., DeAngelo D.J., Pollyea D.A., Sekeres M.A., Robak T. (2018). Glasdegib in combination with cytarabine and daunorubicin in patients with AML or high-risk MDS: Phase 2 study results. Am. J. Hematol..

[42] Kent A., Vasu S., Schatz D., Monson N., Devine S., Smith C., Gutman J.A., Pollyea D.A. (2020). Glasdegib as maintenance therapy for patients with AML and MDS patients at high risk for postallogeneic stem cell transplant relapse. Blood Adv..

[43] DeAngelo D.J., Spencer A., Bhalla K.N., Prince H.M., Fischer T., Kindler T., Giles F.J., Scott J.W., Parker K., Liu A. (2013). Phase Ia/II, two-arm, open-label, dose-escalation study of oral panobinostat administered via two dosing schedules in patients with advanced hematologic malignancies. Leukemia.

[44] Bug G., Burchert A., Wagner E.M., Kröger N., Berg T., Güller S., Metzelder S.K., Wolf A., Hünecke S., Bader P. (2017). Phase I/II study of the deacetylase inhibitor panobinostat after allogeneic stem cell transplantation in patients with high-risk MDS or AML (PANOBEST trial). Leukemia.

[45] Jamy O., Diebold K., Davis K., Bachiashvili K., Rangaraju S., Vachhani P., Godby K.N., Salzman D., Bhatia R. (2023). Impact of induction intensity and transplantation on outcomes of patients with complex karyotype and TP53-mutated acute myeloid leukemia. Bone Marrow Transpl..

[46] Mishra A., Tamari R., DeZern A.E., Byrne M.T., Gooptu M., Chen Y.B., Deeg H.J., Sallman D., Gallacher P., Wennborg A. (2022). Eprenetapopt Plus Azacitidine After Allogeneic Hematopoietic Stem-Cell Transplantation for TP53-Mutant Acute Myeloid Leukemia and Myelodysplastic Syndromes. J. Clin. Oncol..

[47] Nayak R.K., Chen Y.B. (2022). Maintenance therapy for AML after allogeneic HCT. Front. Oncol..

[48] Liga M., Triantafyllou E., Tiniakou M., Lambropoulou P., Karakantza M., Zoumbos N.C., Spyridonidis A. (2013). High alloreactivity of low-dose prophylactic donor lymphocyte infusion in patients with acute leukemia undergoing allogeneic hematopoietic cell transplantation with an alemtuzumab-containing conditioning regimen. Biol. Blood Marrow Transpl..

[49] Schmid C., Labopin M., Schaap N., Veelken H., Schleuning M., Stadler M., Finke J., Hurst E., Baron F., Ringden O. (2019). Prophylactic donor lymphocyte infusion after allogeneic stem cell transplantation in acute leukaemia—a matched pair analysis by the Acute Leukaemia Working Party of EBMT. Br. J. Haematol..

[50] Jedlickova Z., Schmid C., Koenecke C., Hertenstein B., Baurmann H., Schwerdtfeger R., Tischer J., Kolb H.J., Schleuning M. (2016). Long-term results of adjuvant donor lymphocyte transfusion in AML after allogeneic stem cell transplantation. Bone Marrow Transpl..

[51] Guillaume T., Malard F., Magro L., Labopin M., Tabrizi R., Borel C., Chevallier P., Vigouroux S., Peterlin P., Garnier A. (2019). Prospective phase II study of prophylactic low-dose azacitidine and donor lymphocyte infusions following allogeneic hematopoietic stem cell transplantation for high-risk acute myeloid leukemia and myelodysplastic syndrome. Bone Marrow Transpl..

[52] Devillier R., Calmels B., Guia S., Taha M., Fauriat C., Mfarrej B., Venton G., Vivier E., Olive D., Chabannon C. (2021). Phase I Trial of Prophylactic Donor-Derived IL-2-Activated NK Cell Infusion after Allogeneic Hematopoietic Stem Cell Transplantation from a Matched Sibling Donor. Cancers.

[53] Ciurea S.O., Kongtim P., Soebbing D., Trikha P., Behbehani G., Rondon G., Olson A., Bashir Q., Gulbis A.M., Indreshpal K. (2022). Decrease post-transplant relapse using donor-derived expanded NK-cells. Leukemia.

[54] Al Malki M.M., Keyzner A., Suh H.C., Popat U.R., Dwivedi N., Kothari A.S., Buonomo E., Wang Y., Abelowitz N., Murray J. (2024). TSC-100 and TSC-101, TCR-T Cell Therapies That Target Residual Recipient Cells after Reduced Intensity Conditioning Transplantation, Induce Complete Donor Chimerism with Favorable Prognosis: Early Results of a Phase 1 Trial. Transplant. Cell. Ther. Off. Publ. Am. Soc. Transplant. Cell. Ther..

[55] Reville P.K., Kantarjian H.M., Ravandi F., Jabbour E., DiNardo C.D., Daver N., Pemmaraju N., Ohanian M., Alvarado Y., Xiao L. (2021). Nivolumab maintenance in high-risk acute myeloid leukemia patients: A single-arm, open-label, phase II study. Blood Cancer J..

[56] Davids M.S., Kim H.T., Bachireddy P., Costello C., Liguori R., Savell A., Lukez A.P., Avigan D., Chen Y.B., McSweeney P. (2016). Ipilimumab for Patients with Relapse after Allogeneic Transplantation. N. Engl. J. Med..

[57] Haverkos B.M., Abbott D., Hamadani M., Armand P., Flowers M.E., Merryman R., Kamdar M., Kanate A.S., Saad A., Mehta A. (2017). PD-1 blockade for relapsed lymphoma post-allogeneic hematopoietic cell transplant: High response rate but frequent GVHD. Blood.

[58] Zeiser R., Devillier R., Mico’ M.C., Valcarcel D., Call S., Niederwieser C., Nourry C., Xu Y., Medts T., Guichard N. (2023). TIM-3 Inhibitor Sabatolimab for Patients with Acute Myeloid Leukemia (AML) with Measurable Residual Disease (MRD) Detected after Allogeneic Stem Cell Transplantation (AlloSCT): Preliminary Findings from the Phase Ib/II Stimulus-AML2 Study. Blood.

